# Insights into the cellular function and mechanism of action of quinone reductase 2 (NQO2)

**DOI:** 10.1042/BCJ20240103

**Published:** 2025-03-11

**Authors:** Faiza Islam, Brian Shilton

**Affiliations:** Department of Biochemistry, University of Western Ontario, London, Ontario N6A 5C1, Canada

**Keywords:** flavonoids, flavin redox switch, kinase inhibitor, redox homeostasis, redox signalling, quinone reductase

## Abstract

Quinone reductase 2 (NQO2) is a FAD-linked enzyme that cannot use the common reducing cofactors, NADH and NADPH, for efficient catalysis. This is unusual for an oxidoreductase, particularly since it is a member of a large family of enzymes that all use NAD(P)H efficiently to catalyse the two-electron reduction in quinones and other electrophiles. The inability of NQO2 to use NAD(P)H efficiently raises questions about its cellular function: it remains unclear whether the main cellular role of NQO2 is the catalytic reduction in quinones or whether it is a pseudo-enzyme with other roles such as cell signalling. Intriguingly, NQO2 has been identified as an off-target interactor with over 30 kinase inhibitors and other drugs and natural products. The interaction between NQO2 and kinase-targeted drugs is particularly intriguing because it suggests that NQO2 may be contributing to the cellular effects of these drugs. In this review, we will discuss the enzymatic properties of NQO2, its structure and complexes with various drugs and small molecules, potential cellular roles, and some of the enigmatic findings that make this molecule so interesting and worthy of further investigation.

## Introduction

Human quinone reductases 1 and 2 (NQO1 and NQO2) are members of an ancient family of quinone reductase (QR) enzymes, which are flavin-dependent oxidoreductases that catalyse the obligate two-electron reduction in quinones and other electrophiles. NQO1 and NQO2 are paralogs with 48% sequence identity and similar structures. However, the two proteins have key differences, suggesting different cellular functions. NQO1 has evolved to be an excellent catalyst for the reduction in quinones or other electrophiles, using NAD(P)H as reducing co-substrates. As part of the Keap1/Nrf2 pathway, NQO1 has enzymatic roles in response to oxidative stress [1]. In contrast, NQO2 has evolved to be highly catalytically inefficient using NAD(P)H as reducing co-substrates such that its ability to catalyse quinone reduction *in vitro* is barely detectable [2,3]. On this basis, the cellular roles of NQO2 are not well defined, and the inability of NQO2 to efficiently use NAD(P)H as reducing co-substrates brings into question its potential for enzymatic reduction in quinones or other electrophiles [4].

NQO2 can efficiently use smaller natural or synthetic dihydronicotinamide co-substrates, such as dihydronicotinamide riboside (NRH) or the synthetic 1-benzyl-1,4-dihydronicotinamide (BNAH) [5,6]. This is not a unique feature of NQO2, as NQO1 can use smaller dihydronicotinamide cofactors with moderately high efficiency [3,7]. Despite the ability of NQO2 to efficiently use NRH, it has been repeatedly shown that NQO2 in cells is unable to catalytically reduce a toxic electrophile, CB1954, without the addition of exogenous small dihydronicotinamide cofactors such as NRH or BNAH [2,6]. Thus, the cellular levels of NRH appear to be too low to support significant catalysis by NQO2. A low level of available NRH in cells is consistent with observations that exogenous NRH is fed into salvage pathways that ultimately lead to large increases in cellular NAD^+^ levels [8,9]; that is, cells do not seem to maintain a significant pool of NRH but, instead, convert it to NAD^+^. In summary, the apparently low levels of cellular NRH mean that NQO2 has effectively lost the ability to efficiently catalyse reduction in quinones or other electrophiles in cells.

Although it cannot use NAD(P)H efficiently, NQO2 has retained the capability to exist in an oxidized or reduced state and could function in cellular signalling and regulation. Furthermore, the unusual catalytic properties of NQO2 have been conserved throughout the amniotes over millions of years of evolution, consistent with selective pressure for cellular functions that require these properties [2]. Regarding possible functions, there is evidence that NQO2 affects the cellular response to oxidative stress, has roles in learning and memory, and interacts with many bioactive compounds, including melatonin, resveratrol, and over 30 kinase inhibitors.

In this review, we aim to synthesize the current advances in understanding the cellular function of NQO2, particularly with respect to possible roles in responding to the redox landscape to regulate metabolism and cell survival pathways involving apoptosis and autophagy. Additionally, we discuss the question at the heart of the problem on NQO2 cellular function – does it function as a QR like NQO1, or did it evolve to have a novel signalling function?

The review is divided into two broad sections – the first concerns the proposed cellular functions of NQO2 and the second discusses proposed mechanisms of NQO2 action in cells. We highlight a hypothesis on the mechanism of action of NQO2 as a flavin redox switch, involving signalling based on the ratio of redox couples in cells.

## The cellular functions and effects of NQO2

### A role for NQO2 in memory and learning

NQO2 is involved in regulating memory and learning behaviours in mammalian brains. A potential role for NQO2 in neurological function started with the observation that NQO2 transcript levels are significantly up-regulated in two different mouse models for memory function. In the first model, memory-impaired aged mice had higher levels of NQO2 transcripts than their non-impaired counterparts [10]. A second study using a pharmacological (scopolamine-induced) amnesia mouse model also showed greater NQO2 transcripts in memory-impaired mice [11]. These studies were followed up by the same group, who used NQO2-knockout mice or S29434, a specific NQO2 inhibitor [12], to show that the absence or inhibition of NQO2 improved learning behaviours [13]. The group detected the NQO2 protein in the cortex and hippocampus, regions of the brain involved in learning and memory.

Results demonstrating a function for NQO2 in learning and memory have been recapitulated and extended by Rosenblum and colleagues [14–17]. In line with previous observations, they showed that NQO2-knockout mouse models performed significantly better on memory and learning tests than control mice and that inhibition of NQO2 with S29434 ameliorated the memory performance of an amnesia mouse model [14]. Using a novel taste learning test in rats to investigate memory, Rosenblum’s group observed that NQO2 transcript levels were 50% down-regulated in the cortex after a novel taste experience. This decrease in NQO2 transcript levels was shown to be regulated by the muscarinic-acetylcholine receptor (mAChR) signalling pathway for memory consolidation. That is, scopolamine-induced disruption of mAChR signalling inhibited NQO2 transcriptional down-regulation. Consistent with a role for decreased NQO2 in learning, directly decreasing NQO2 transcript levels by shRNA enhanced novel taste learning in the animal models [14,15]. The same down-regulation of NQO2 also occurred in neurons of the hippocampus in rodent models during learning [16]. They further showed that NQO2 removal or its enzymatic inhibition with S29434 was able to reduce inhibitory neuronal excitability as evidenced by a significant reduction in neuronal firing and a hyperpolarized resting membrane potential [17]. The group concluded that NQO2 is a previously unknown member of the canonical mAChR signalling pathway for learning.

### Regulation of cell death in response to oxidative stress

NQO2 affects the cellular response to redox stress in a manner that is not consistent with the enzymatic reduction in quinones. This is demonstrated by the response of NQO1- or NQO2-knockouts to oxidative challenges by menadione, a quinone substrate for both enzymes: NQO1-knockout mice exhibited increased sensitivity to menadione [18], while NQO2-knockout mice showed decreased sensitivity to menadione [19]. The increased sensitivity of the NQO1-knockouts is consistent with a role for NQO1-mediated two-electron reduction in menadione, which is expected to reduce oxidative stress; the absence of NQO1, therefore, makes the cells more sensitive. On the other hand, the *decrease* in menadione sensitivity of the NQO2-knockouts is not compatible with the role of catalytic quinone reduction by NQO2. On the contrary, the presence of NQO2 exacerbates the toxicity of menadione, an effect that is even stronger when a suitable exogenous dihydronicotinamide co-substrate is supplied. These results were reinforced by experiments in Chinese hamster ovary (CHO) cells, where the overexpression of NQO2 increased the toxicity of menadione compared with wildtype cells, and the menadione toxicity was further increased when it was administered in the presence of NRH [20]. These results suggest that NQO2 is not reducing the menadione to alleviate cellular oxidative stress but is instead responding to oxidative stress by promoting apoptosis. Thus, in NQO2 knockouts, the apoptotic response to oxidative stress appears to be down-regulated.

The involvement of NQO2 in a downstream cellular response to oxidative stress is supported by the treatment of cells with several oxidative stress inducers. NQO2 inhibition protected astrocytes from paraquat, hydrogen peroxide, and rotenone-induced apoptosis due to redox stress. However, unlike menadione, these stressors were not NQO2 substrates [21]. The same group further showed that NQO2 regulated autophagy in astrocytes upon exposure to paraquat. Paraquat treatment decreased basal autophagy in astrocyte cells, but NQO2 inhibition or knockdown reversed paraquat-mediated autophagy inhibition, suggesting that NQO2 was involved in autophagy regulation [22]. Besides paraquat, a similar NQO2-mediated inhibition of autophagy in astrocyte cells was observed after exposure to 6-hydroxydopamine (6OHDA). 6OHDA is not a substrate of NQO2, but it is suspected to be metabolized in cells to produce potential quinone substrates [23]. It was concluded that NQO2 was involved in the downstream cellular response to redox stress and is a key player in autophagy regulation.

### Target of bioactive compounds and chemotherapeutics

Understanding the cellular function of NQO2 is important as it is an unexpected target of many bioactive compounds and drugs. In Syrian hamster brain tissue, NQO2 was identified as the third binding site for the neurohormone melatonin [24]. Circulating melatonin is produced by the pineal glands and regulates the sleep–wake cycle in concert with the circadian system. The concentrations of melatonin in plasma are low, peaking at about 40 pM [25], and circulating melatonin is primarily thought to affect cell signalling by binding with high affinity to the G-protein coupled receptors, MT1 and MT2, in the brain and a few peripheral organs [26]. NQO2 has a much weaker affinity for melatonin, with a *Kd* of approximately 1 µM [27], making it difficult to understand how the low concentrations of circulating melatonin might affect NQO2. On the other hand, most melatonin is synthesized in extra-pineal tissues and appears to have important functions in redox homeostasis, with much higher concentrations inside cells, particularly within mitochondria [28–31]. A melatonin derivative, 5-methoxy-3-(5-methoxyindolin-2-yl)-1*H*-indole, was developed that binds preferentially to NQO2 with low nanomolar affinity. This compound was shown to be neuroprotective against oxidative stress, similar to melatonin, and it activated neurogenesis from neural stem-cell niches of adult mice; however, it is not known whether the cellular effects were due to its interaction with NQO2 or its antioxidant capacity [32].

NQO2 is a biological target of resveratrol, demonstrated through its consistent avid binding to a resveratrol affinity column [33]. NQO2 binds resveratrol with, by far, the highest affinity (*Kd* = 35 nM) among the other resveratrol targets [34]. Resveratrol has been shown to affect several cellular pathways such as apoptosis, autophagy, cellular metabolism, and redox status [34]. Although there is great interest in its health-related effects, resveratrol binds to many protein targets and can be metabolized to different forms, and so its mechanisms of action are difficult to define [35,36]. Nevertheless, studies with resveratrol have uncovered regulatory pathways in which NQO2 is involved. Resveratrol reduces cell proliferation of vascular smooth muscle cells (VSMCs), while reducing the expression of NQO2; cells treated with siRNA to knockdown NQO2 also showed decreased cell proliferation, demonstrating a correlation between NQO2 levels and VSMC proliferation [37]. Further investigation showed that resveratrol reduced angiotensin-II-induced proliferation of VSMC by disrupting the Ras/MAPK/ERK pathway through decreases in ERK1 and ERK2 phosphorylation, an effect mimicked by siRNA-mediated knockdown of NQO2 [38]. Resveratrol attenuated cell proliferation of androgen receptor-positive, hormone-non-responsive CWR22Rv1 prostate cancer cells [39]. In this case, siRNA knockdown of NQO2 significantly decreased cell proliferation in the absence of resveratrol, and NQO2 knockdown had a small effect on the relative decreases in proliferation in response to resveratrol, supporting the idea that NQO2 was responsible for some of the effects of resveratrol. Further investigation of NQO2-dependent effects in these cells, using stable siRNA knockdown of NQO2, confirmed that the decreases in cell proliferation by resveratrol were partly dependent on NQO2. The NQO2 knockdown attenuated the breakdown of cyclin D1, apparently through a decrease in phosphorylation of cyclin D1 at T286, which was accompanied by a decrease in phosphorylation of retinoblastoma [40]. The NQO2 knockdown was accompanied by an increase in phosphorylation of GSK-3β, suggesting an NQO2-mediated increase in AKT activity [40]. In a subsequent manuscript, an interaction between NQO2 and AKT was characterized by pull-down assays [41]. Although the role of resveratrol is complex, the results indicated that NQO2 is involved in AKT-mediated signalling.

Curcumol was found to overcome tumour necrosis factor(TNF)-related apoptosis-inducing ligand (TRAIL) resistance of non-small cell lung cancer (NSCLC), and NQO2 was identified as an important curcumol target, binding with a *Kd* of 0.58 µM [42]. Curcumol helped to overcome resistance to TRAIL-induced apoptosis by inducing reactive oxygen species (ROS)/endoplasmic reticulum (ER) stress leading to up-regulation of C/EBP homologous protein (CHOP) and death receptor 5 (DR5), which was thought to sensitize cancer cells to TRAIL. The ER stress and up-regulation of CHOP-DR5 appeared to be mediated by an increase in cellular ROS, which was attributed to the interaction between NQO2 and curcumol. The NQO2-dependent effect of curcumol was confirmed by NQO2 knockdown, which increased TRAIL-induced apoptosis [42]; that is, either curcumol binding to NQO2 or NQO2 knockdown sensitized cells to TRAIL-induced apoptosis. A connection between NQO2 and TNF signalling was made over a decade earlier using keratinocytes from NQO2 knockout (NQO2⁻/⁻) mice [43]. In this case, it was shown that in the absence of NQO2, TNF failed to induce expression of NF-κB. The failure of TNF to induce NF-κB also reduced the expression of NF-κB-regulated antiapoptotic genes, MMP-9, cyclin D1, COX-2, IAP1/2, Bcl-2, cFLIP, and XIAP. In the absence of NQO2, the IκB kinases (IKK) were not activated and the degradation of IκBα was abrogated; in addition, there was a lack of activation of signalling pathways involving MAPKs such as JNK, Akt, p38, and p44/p42. These findings suggest that NQO2 supports cell survival by facilitating TNF-induced pro-survival signalling, while its absence sensitizes cells to TNF-induced apoptosis [43].

In addition to a polyphenols such as resveratrol, NQO2 is a target of a variety of flavones, a class of flavonoid compounds characterized by a 2-phenyl-1-benzopyran-4-one core. An early investigation showed that NQO2 was potently inhibited by quercetin, morin, galangin, and chrysin [44]. Notably, NQO2 was not inhibited under the assay conditions by potent NQO1 inhibitors dicumarol, Cibacron Blue, phenidone, and 7,8-dihydroxyflavone, demonstrating that NQO1 and NQO2 can have very different inhibitor binding properties despite their structural similarity. A comprehensive screen of 230 flavonoid-like compounds revealed six sub-micromolar binders of NQO2, namely, chrysoeriol, 5-hydroxyflavone, kaempferol, apigenin, luteolin, and isorhamnetin, along with 14 compounds that bound to NQO2 with low micromolar affinity [45]. Flavonoids can have a number of cellular effects, particularly in autophagy and apoptosis [46] that may be relevant to NQO2 cellular function [12,22]. Seven flavonoids that inhibit NQO2 with IC50 values ranging from 0.15 µM to 80.5 µM were tested for their ability to induce autophagy, assayed by LC3 lipidation in HepG2 cells [47]. Submicromolar IC50 inhibitors of NOQ2, namely apigenin, luteolin, and quercetin, showed the highest induction of autophagy. Genetic silencing of NQO2 slightly augmented basal autophagic signal but reduced the autophagy response to apigenin and luteolin, the two flavonoids tested. Both luteolin and apigenin stimulated AMPK phosphorylation, which was linked to autophagy activation. Silencing of NQO2 caused an increase in basal phosphorylation of AMPK but abrogated the increased phosphorylation effected by luteolin and apigenin [47].

NQO2 is also a very frequent off-target interactor of numerous chemotherapeutic kinase inhibitor drugs [48,49]. A large-scale screen involving 234 clinical kinase inhibitors identified NQO2 as a binding target for 32 drugs, with a sub-micromolar affinity for 10 drugs (Table 1) [48]. Structural studies with imatinib, tetrabromo inhibitors of CK2, crenolanib, volitinib, and pacritinib, among others, indicate that drugs bind to the NQO2 active site by stacking on top of the isoalloxazine ring of the FAD cofactor with additional interactions mediated by surrounding protein residues [48,74,75]. It is noteworthy that NQO1 was not identified as an off-target interactor with any of the 234 inhibitors. Clinical kinase inhibitors may leverage promiscuity to disrupt multiple oncogenic pathways to be efficacious. For example, imatinib, designed for BCR-Abl kinase inhibition, also targets PDGFR, contributing to its therapeutic effects [74]. Some kinase inhibitors have also been shown to exert anticancer effects through unintended non-kinase targets [75]. Tivantinib, initially aimed at c-MET kinase inhibition, induced cytotoxicity independent of c-MET inhibition. Instead, binding to the non-kinase target, tubulin, seemed to contribute to tivantinib efficacy. Tubulin is a frequent non-kinase off-target interactor of kinase inhibitors. Tubulin polymerization is required to form microtubules, which, in turn, are crucial for mitosis in rapidly dividing cancer cells, so tubulin polymerization inhibition is cytotoxic to cancer cells [76,77]. Given that the interactions of NQO2 with melatonin, resveratrol, curcumol, and flavonoids affect cellular stress responses that can involve kinase-mediated signalling pathways, the off-target interactions of NQO2 with kinase-targeted inhibitors could contribute to their therapeutic effects.

**Table 1:** Clinical kinase inhibitors that bound to NQO2, alongside their intended molecular targets.

Drug	Main or intended molecular target	Total number of protein targets	EC_50_ for NQO2 (nM)^1^	EC_50_ for target (nM)^1^	IC_50_ for target (nM)^2^
AMG-900	AURKB, AURKA, AURKC	39	3353	34, 19, ND^4^	5, 4, 1 [50]
ARRY-380	HER2	3	2463	ND^4^	8 [51]
ASP-3026	ALK	39	6362	ND^4^	3.5 [52]
Binimetinib	MAP2K1, MAP2K2	3	14,700	45, 36	12 (for both) [53]
BMS-911543	JAK2	1	5864	ND^4^	1.1 [54]
CC-401	MAPK8, MAPK9, MAPK10	26	1413	ND^4^	K_i_ 25–50 [55]
Crenolanib	FLT3, PDGFRA, PDGFRB	69	40	33, ND^4^, 121	K_D_ 0.74, 2.1, 3.2 [56]
CYC-116	AURKB, AURKA	111	648	115, 334	8, 9 [57],
Dovitinib	multi-kinase	73	3011		
Fasudil	ROCK1, ROCK2	20	2450	996, 1082	330, 160 [58]
Fedratinib^3^	FLT3, JAK2	35	4314	1246, ND^4^	15, 3 [59]
Gilteritinib**^3^**	FLT3, ULK3	82	1701	ND^C^, 14	0.29 [60]
Imatinib**^3^**	ABL, PDGFRA, PDGFRB, DDR1	15	41	194, ND^4^, ND^4^, 35	
JNJ-38877605	MET	3	323	3	4 [61]
K-252A	multi-kinase	106	4030		
KW-2249	FLT3	72	3597	69, 3757, 202	6.6 [62]
Lenvatinib**^3^**	VEGFR2, VEGFR3	19	1522	ND^4^, ND^4^, 20	4, 5.2 [63,64]
Masitinib	KIT, PDGFRA, PDGFRB	10	470	ND^4^, 5426	200,540,800 [65]
Nilotinib**^3^**	ABL1	15	1484	550	
OSI-027	mTORC1, mTORC2	12	1390	ND^4^, ND^4^	22, 65 [66]
Pacritinib**^3^**	JAK2, FLT3	42	4	ND^4^, 424	23, 22 [67]
Pictilisib	P13KCA, PIK3CD	13	1027	886	3 [68]
RDEA-436	MAP2K1, MAP2K2	7	370	72, 27	
Rigosertib	PLK1	4	15,224	ND^4^	
Ripasudil	ROCK1, ROCK2	18	3699	37, 95	19, 51 [69]
SCH-900776	CHEK1	21	2359	13	3 [70]
SGX-253	MET	1	125	ND^4^, 4.16	4 [71]
Sunitinib**^3^**	VEGFR, PDGFRB	100	4022	ND^4^, 4.16	
Talmapimod	Multi MAP kinases	2	9918	16	
Tepotinib**^3^**	MET	2	875	1	3 [72]
Volitinib	MET	1	206	ND^4^	5 [73]

1 The EC50 was determined from the Kinobead elution gradient; data accessed at www.proteomicsdb.org, project Klaeger_Science_2017 ID PRDB004257.

2 Sources for the IC50 values for protein targets are indicated by references in parentheses.

3 These drugs are FDA-approved kinase inhibitors that are being used in the clinic.

4 These drugs did not pick up their known targets in the screen so there is no apparent affinity.

**Figure 1:**
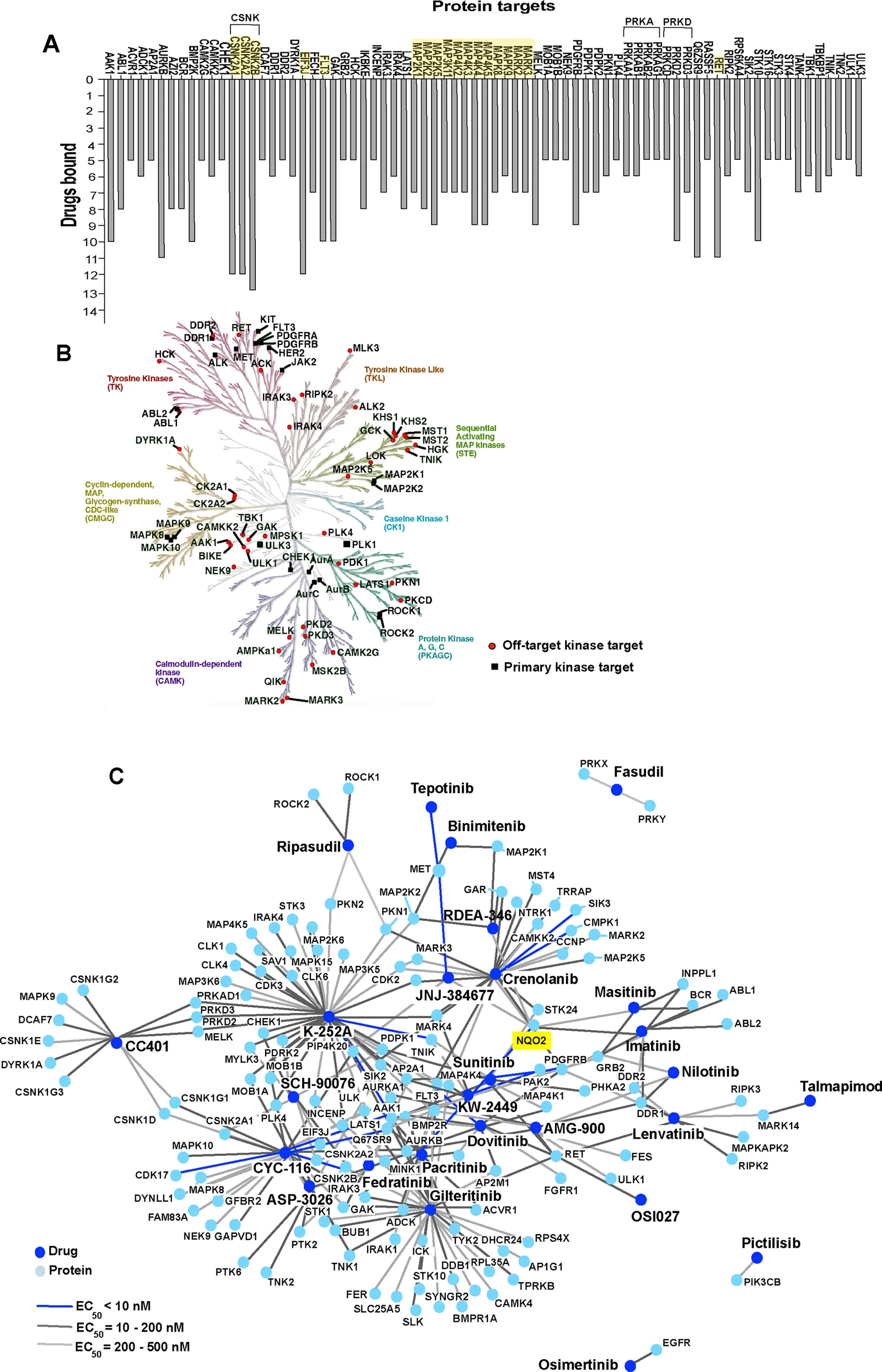
Interaction of NQO2 with clinical kinase inhibitors. Out of 232 clinical kinase inhibitors, NQO2 was an off-target interactor with 32, and it bound to at least 10 drugs with sub-micromolar affinity. We analysed the other protein interactors of the 32 drugs that bound to NQO2 to ascertain if there were other common proteins bound by those drugs that could function in pathways in concert with NQO2. (**A**) The common protein targets of the 32 NQO-interacting drugs are arranged in alphabetical order. Because of their promiscuity for various kinases, the 32 NQO2-interacting drugs bound to 207 other protein interactors. Shown in the bar graph are proteins that bound to five or more of the NQO2-interacting drugs: 51 kinases along with 13 non-kinase proteins. The highlighted protein targets are discussed in the text. Note that for Casein Kinase 2 (CSNK), Protein Kinase A (PRKA), and Protein Kinase D (PRKD), multiple catalytic and/or non-catalytic subunits were identified by the same drugs. (**B**) The 32 drugs that bound to NQO2 were promiscuous in their affinity for several different kinases. Shown is the phylogenetic tree of the kinome [78] with the 51 kinase targets of the 32 drugs shown in A. The kinases annotated in black were the main intended (primary) targets of the drugs while the ones in red were the off-target interactors. (**C**) Interaction plot showing other protein targets of the 32 drugs that bound to NQO2 for interactions up to an EC_50_ cut-off of 0.5 μM. The plot shown has 27 of the total 32 drugs (shown in dark blue) as the other drugs only had targets of lower affinity than the 0.5 μM cut-off. The highest affinity interactions (EC_50_<10 nM) are shown by connecting lines in blue; EC_50_ between 10 nM and 200 nM in dark grey; and EC_50_ between 200 nM and 500 nM in light grey. NQO2 (highlighted) is bound to seven of the drugs with the specified affinities. The list of the 234 clinical kinase inhibitors tested by Klaegar et al. [48] was retrieved from the manuscript, and further data on interactions of these drugs with proteins were retrieved from https://www.proteomicsdb.org/, from the project Klaegar_Science_2017, ID PRDB004257, where each of the 234 clinical kinase inhibitors tested were searched individually to find listed protein interactors and their respective affinities. NQO2, human quinone reductase 2.

We looked further into the 32 NQO2-interacting kinase inhibitors to assess their protein targets to find common cellular signalling pathways that could illuminate the possible cellular and/or synergistic effects elicited by NQO2 drug binding. The main intended targets of these drugs (Table 1) were predominantly tyrosine kinases (Figure 1B), likely because the tyrosine kinases were identified as optimal clinical chemotherapeutic targets. At least four of these inhibitors were intended to be multi-kinase inhibitors and they bound to hundreds of proteins. However, most of the 32 drugs that bound to NQO2 were much more selective. Notably, NQO2 bound to four c-MET receptor kinase-specific inhibitors, SGX-253, Volitinib, JNJ-38877605, and Tepotinib. These inhibitors appeared to be relatively selective as in the screen they only bound to the MET kinase and NQO2 with sub-micromolar affinity, suggesting that NQO2 inhibition may affect the mechanism of action of these drugs. SGX-253 and JNJ-38877605 had to be discontinued for clinical trials due to unexpected renal toxicity [79,80]. Volitinib is being used clinically for the treatment of metastatic NSCLC in China. Tepotinib is US FDA-approved for the treatment of patients with metastatic NSCLC with a MET mutant variant. Another common main target of five drugs, namely, Crenolanib, Pacritinib, Fedratinib, Gilteritinib, and KW-2249, was FLT3. NQO2-interacting drugs also frequently targeted the kinases PDGFR, JAK2, and BCR-Abl. Notably, these kinases are all involved in the RAS/MAPK, P13K/AKT, and JAK2/STAT pathways.

Most of the NQO2-interacting drugs bound to numerous other kinase and non-kinase proteins; the numbers of interactions are listed in Table 1. To understand the cellular signalling pathways that might be affected by drug binding to NQO2, we also looked at other common adventitious targets of the inhibitors (Figure 1A and B). We analysed the list of proteins that were shown to bind to at least 5 of the 32 drugs that bound to NQO2. The most frequent target of these drugs was CSNK2 (otherwise known as kinase CK2) with at least six drugs binding with an apparent sub-micromolar affinity. CK2-targeted inhibitors TBBZ and DMAT were first identified in off-target interactions with NQO2 in 2008 [81], which was followed by a comprehensive binding and structural analysis [82]. Besides CK2, another top common adventitious target that bound to six drugs with sub-micromolar affinity was EIF3J, or the Eukaryotic Translation Initiation Factor 3 subunit J, which is a putative CK2 substrate [83]. It was shown that CK2-mediated phosphorylation of EIF3J is essential to promote the assembly of the EIF3 complex and activate the translation initiation machinery [83]. It is also possible that the screen picked up CK2 and EIF3J as a complex together as these two proteins were identified with the same drugs (Figure 1C). It is noteworthy that another class of frequently targeted proteins were several MAP kinases such as MAP2K1, MAP2K2, MAP2K5, MAP3K11, and MAP4K1-5. MAP2K1 and MAP2K2 are involved in directly activating ERK1/ERK2 in the RAS/RAF/MAPK signalling pathway. Another protein frequently identified as a target by these drugs was the RET kinase, which is often aberrant in NSCLC and thyroid carcinoma. RET is a transmembrane receptor tyrosine kinase and it activates P13K/AKT, RAS/RAF/MAPK, JAK2/STAT3, and PLCγ [84]. Additionally, FLT3 was also a frequent adventitious target of these kinase inhibitors. FLT3 is also involved in feeding into the RAS/RAF/MAPK and P13K/AKT pathway. This analysis also showed that the RAS/MAPK pathway was particularly enriched, as many upstream kinase receptors and the MAPK kinases were targets for the inhibitors.

Our analysis of NQO2-interacting kinase inhibitors suggested that NQO2 may be synergizing with the RAS/MAPK pathway, which transduces signals for cell growth, division, and differentiation. Overall, NQO2 appears to have intriguing functions in learning and memory, regulation of cell metabolism, proliferation, and death in response to changes in the redox landscape. These functions, illuminated through binding of kinase inhibitors or xenobiotics, or silencing of NQO2, are difficult to attribute to the catalytic reduction in quinones. In the next section, we will discuss potential mechanisms for NQO2 cellular function.

## Proposed mechanism of NQO2 function

### Evolution of NQO2 points to non-catalytic functions

**Figure 2:**
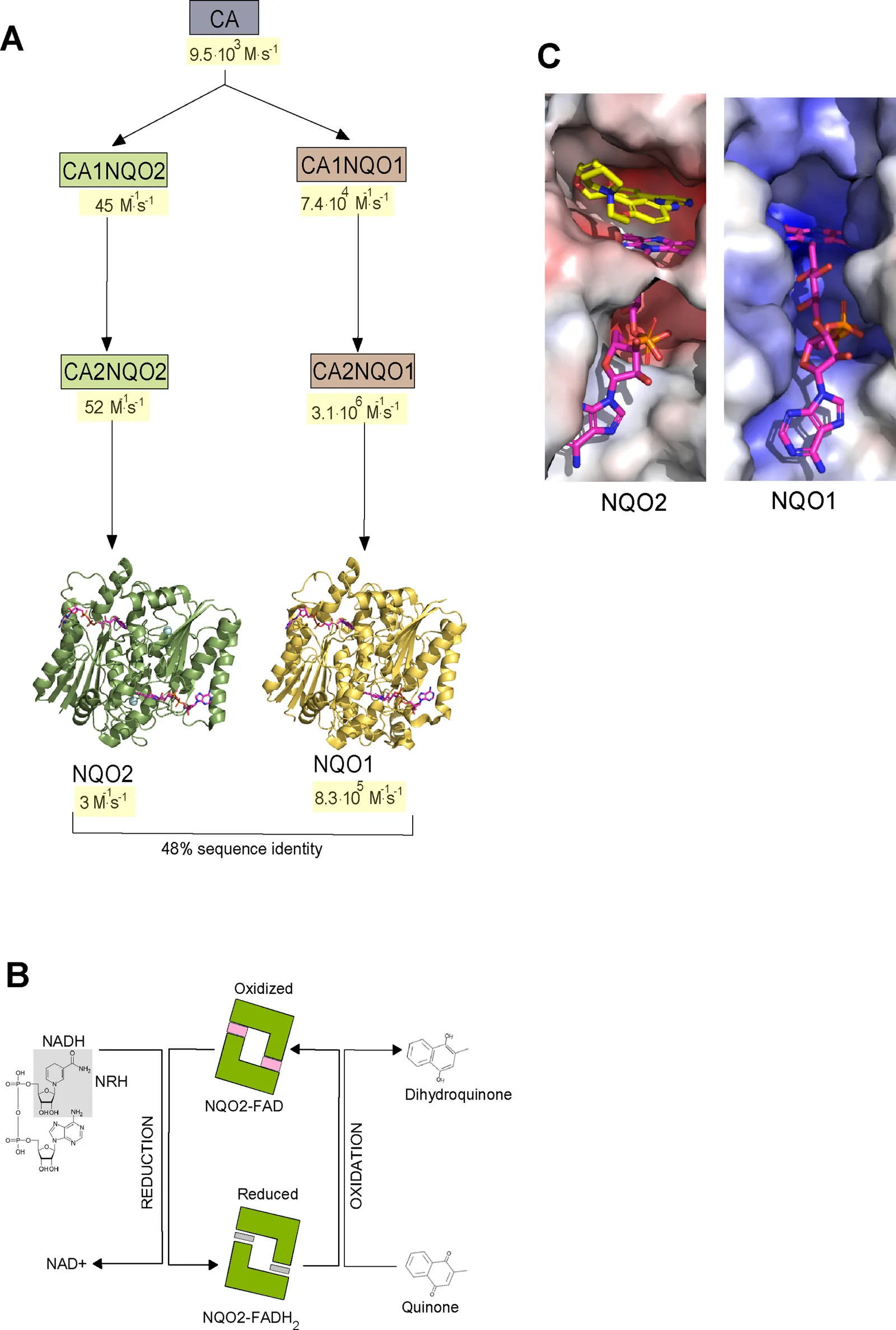
Function and structure of quinone reductases NQO1 and NQO2. (**A**) Evolution of NQO1 and NQO2. Ancestral state reconstruction was used to track the evolution of NQO1 and NQO2 in terms of their catalytic efficiencies (yellow highlights) with NADH as a reducing co-substrate [3]. The common ancestor (CA) exhibited moderate efficiency with NADH, which was further increased along NQO1 lineage, from CA1NQO1 to CA2NQO1 and finally extant NQO1. In contrast, the catalytic efficiency with NADH drastically decreased along the NQO2 lineage. It is noteworthy that all the enzymes are able to use NRH or the synthetic dihydronicotinamide cofactor BNAH with moderate to high catalytic efficiency, and there was no systematic change in catalytic efficiency over time in this regard. (**B**) The redox cycle of NQO2 and related quinone reductases. In the initial reductive half-reaction, NQO2 binds a reducing co-substrate (NAD(P)H or NRH) and the FAD cofactor undergoes a 2-electron reduction to FADH_2_. Then, in the oxidative half-reaction, the reduced form of the protein transfers the 2 electrons from FADH_2_ to the quinone substrate to produce the dihydroquinone product and the protein is returned to its oxidized form. Both NQO1 and NQO2 can efficiently use NRH as a reducing co-substrate, but NQO2 cannot use NAD(P)H efficiently. (**C**) The active sites of NQO1 and NQO2 are structurally similar, consisting of the FAD cofactor bound in a cavity formed between the two protomers of the homodimer. The electrostatic potential of the active site of the two proteins differs as NQO2 has a neutral (white) to slightly negative (red) active site, while NQO1 has a positive active site (blue). Despite the structural similarity, only NQO2 was found to bind clinical kinase inhibitors, that typically stack on top of the isoalloxazine ring of the FAD cofactor [48]. Shown is the structure of the active site of NQO2 bound to the kinase inhibitor pacritinib (yellow carbons). The protein structures of NQO1 and NQO2 were accessed from the Protein Data Bank (https://www.rcsb.org/) with codes 1D4A and 5LBZ, respectively [48,85]. NQO1, human quinone reductase 1; NQO2, human quinone reductase 2.

A central issue concerning NQO2 cellular function is whether it catalyses reduction in quinones and other electrophiles in cells or whether it functions in a non-catalytic manner in cell signalling. Unlike NQO1 and other QR enzymes, NQO2 has evolved to use NAD(P)H inefficiently, to the extent that it cannot effectively catalyse reactions with NAD(P)H. The highly inefficient use of NAD(P)H by NQO2 is conserved across the amniotes [2], indicating that this property was subject to natural selection and, therefore, has functional significance. When NQO2 was first purified, it was suggested that its function was to consume cellular NRH [5], and this has led to the idea that NQO2 evolved to use NRH or another reductant for catalysis. However, ancestral sequence reconstruction and analysis of extant NQO1 and NQO2 showed that the two proteins underwent functional divergence after they evolved from the duplication of a common ancestral gene [3]. The evolutionary analysis clearly shows that throughout their evolution, both NQO1 and NQO2 could use NRH efficiently, but NQO2 specifically evolved to lose its catalytic activity with conventional reducing co-substrates, NAD(P)H. The fact that the enzymes, beginning with the common ancestor, can all use NRH efficiently presents a strong argument that NQO2 has evolved specifically not to use NAD(P)H efficiently (Figure 2A). Consistent with this observation, the available evidence indicates that there is not sufficient NRH, or any other reducing co-substrate, to support the catalytic reduction in electrophiles by NQO2 in cells [2,6]. On the other hand, NQO2 did not lose its ability to bind the FAD cofactor and can exist in either an oxidized or reduced state [3]. This has led us to hypothesize that NQO2 may respond in a redox-sensitive manner to the relative levels of NAD(P)^+^ and NAD(P)H in cells. Unlike an enzyme, whose primary function is to turnover substrate into product, a signalling protein may exploit poor enzymatic efficiency as a signalling strategy.

Another QR that evolved to have a non-catalytic role is the yeast Lot6p. In yeast, Lot6p was shown to stabilize the transcription factor, Yap4, in a redox-sensitive manner, to protect it from association with the yeast 20S proteasome [86] and induce apoptosis [87]. Notably, Lot6p reduction in electrophiles is very slow, and it was speculated that it may have evolved this property as a signalling strategy [88]; that is, after Lot6p reduction by NADH, it will bind to Yap4, and slow re-oxidation by electrophiles will prolong the duration of the Yap4-binding signal. Similarly, the inefficient and slow reduction in NQO2 by NAD(P)H may have a functional role in regulated redox signalling.

### NQO1 mechanism – catalytic and non-catalytic roles

Given the high degree of similarity between NQO1 and NQO2, mechanisms of NQO1 function may provide insight into NQO2. In addition to its role in the catalytic reduction in quinones, NQO1 has been shown to moonlight as a regulator of protein degradation by binding to proteins and regulating their interaction with the 20S proteasome [89]. The 20S proteasome catalyses ubiquitin-independent degradation of proteins with intrinsically unstructured or unfolded regions [90], and NQO1 was shown to protect p53 from proteasomal degradation [91,92]. Both NQO1 and NQO2 appear to link cellular metabolism to cell survival by stabilization of p53 in response to inhibition of mitochondrial complex III [93]. In this case, pharmacological interventions disturbed mitochondrial activity, which led to stalling of *de novo* pyrimidine synthesis, induced p53 accumulation, and subsequent cellular apoptosis. Treatment with inhibitors or knockdown of NQO1 and NQO2 was correlated with reduced stabilization of p53. Besides p53, NQO1 has been shown to stabilize p33 [94], p73 [95], ornithine decarboxylase [96], HIF1α [97], c-Fos [98], and ELF4G1 [99].

### NQO2 as a flavin redox switch

NQO2 has lost the capacity for efficient catalysis using NAD(P)H, but the FAD cofactor is fully functional so that NQO2 can still exist in either an oxidized or reduced state and, on this basis, can have non-catalytic redox functions (Figure 2B). One possibility is that NQO2 can propagate signals about fluctuating NAD(P)H/NAD(P)^+^ levels by functioning as a flavin redox switch. In this class of flavoproteins, a change in the redox state of the protein-bound FAD cofactor is converted into a new functional output primarily through structural changes in the protein [100]. An example of a flavin redox switch is the NIFL flavoprotein that sensitizes the transcriptional activation of nitrogen-fixing genes with the redox status of nitrogen-fixing bacteria. Mechanistically, the oxidized form of NIFL binds to NIFA and blocks its transcriptional activation, but once the flavin cofactor is reduced, the protein structure changes and no longer blocks NIFA [101].

Previously, our group showed that NQO2 has the potential to function as a flavin redox switch. Reduction in the FAD favours the binding of the antimalarial drug, chloroquine (CQ), and binding of chloroquine to reduced NQO2 leads to a small bend in the isoalloxazine ring that promotes a global conformational change and alters protein–protein interactions [102]. Crystals of oxidized NQO2 were treated with a reducing agent and chloroquine, and diffraction data from the treated crystals were collected. It was clear that the crystals had undergone a marked structural transition, with the unit cell shrinking by 1 to 2 Å in each direction and the space group changing from orthorhombic (P2_1_2_1_2_1_; typical for oxidized NQO2) to lower symmetry monoclinic (P2_1_). At the molecular level, reduction and chloroquine binding caused the NQO2 dimers in the crystal to change their relative orientation and pack more closely together. While there were no large, rigid-body domain movements in the NQO2 dimers, there were localized changes distributed on the surface of the protein that were sufficient to alter the interactions between NQO2 dimers [102]. The binding of chloroquine to *oxidized* NQO2 does not produce a conformational change in the protein, and oxidized NQO2 invariably crystallizes in the same orthorhombic space group, regardless of the bound ligand. Thus, the NQO2-FAD-CQ (oxidized) complex has a different overall structure than the NQO2-FADH2-CQ (reduced) complex, which would enable the oxidized and reduced forms of NQO2 to interact with different proteins in signalling pathways and relay information about the changing redox and metabolic landscape of the cell.

A redox switch mechanism for NQO2 has interesting consequences for the kinase inhibitors that interact with NQO2. Small molecule inhibitors bind to NQO2 by stacking against the isoalloxazine ring of the protein (Figure 2C) and so are often selective for either the reduced or the oxidized state of NQO2. This selectivity is largely due to electrostatic interactions, as reduced FAD has an overall negative charge so that compounds with a more positive electrostatic potential bind preferentially to reduced NQO2 [82,102]. Selective drug binding to either the reduced or oxidized form of a flavoprotein has important implications for a redox switch function since the bound drug will stabilize its preferred form of the protein, effectively shifting the redox equilibrium. This mechanism has been demonstrated for NQO1 with dicoumarol, which binds selectively to the oxidized form of NQO1, making the flavin reduction potential more negative and shifting the overall redox equilibrium further towards the oxidized form [103]. In the case of NQO2, its interactions with kinase inhibitors will affect its redox equilibrium depending on whether the inhibitor binds selectively to either the reduced or oxidized form. A shift in the redox equilibrium of NQO2 effected by the binding of a kinase inhibitor will alter the response of NQO2 to the cellular NAD(P)H/NAD(P)^+^ ratio and hence the signal that is propagated.

### NQO2 and changes in ROS

A number of groups have independently shown that functional NQO2 is associated with increases in cellular ROS [21–23,38,104–107], and changes in cellular ROS could be involved in NQO2-mediated signal transduction. In the brain, Rosenblum showed that NQO2 functions as a memory constraint that is down-regulated after a novel taste learning experience. This was initially observed as a decrease in NQO2 mRNA expression after the learning experience. In addition, experimental decreases in NQO2, either using an inhibitor (S29434) or shRNA knockdown, enhanced novel taste learning. An association of NQO2 with cellular redox regulation was observed when young and aged mice were compared: the aged mice with poor memory had increased levels of NQO2 and increases in oxidized (oligomerized) Kv2.1 potassium channels [16]. The change in the Kv2.1 channel oxidation was also observed in another study, along with apparent changes in cellular ROS measured indirectly using a fluorescent reporter [15].

ROS are produced by oxidative phosphorylation. A recent manuscript by Rosenblum’s group in collaboration with our lab showed that NQO2 may contribute to the regulation of cellular metabolism [108]. Knockouts of NQO2 in a colon cancer cell line (HCT116-NQO2Δ) exhibited distinct proteomic changes: increased expression of mitochondrial proteins involved in mRNA translation and oxidative phosphorylation coupled with decreases in proteins involved in glycolysis and the pentose phosphate pathway. HCT116-NQO2Δ cells also showed increased expression of proteins related to mRNA transcription and translation, and decreased expression of proteins related to cell–cell junctions and cell–matrix interactions. The HCT116-NQO2Δ cells showed decreased baseline levels of ROS. Overall, the roles of NQO2 in memory consolidation and learning appear to be linked to changes in cellular metabolism and/or ROS species.

Additional studies of NQO2-mediated changes in cellular ROS are listed in Table 2. Typically, cells were treated with a redox stressor to stimulate ROS, and then modulation of NQO2 by binding of an inhibitor (S29434) or genetic knockdown techniques decreased ROS. NQO2-mediated changes in ROS have been measured primarily with the fluorescent probes, 2′,7′-dichlorodihydrofluorescein diacetate (H_2_DCFDA), and a dihydroethidium derivative, mitochondria-targeted probe, MitoSox, in a range of cell types. MitoSox is selective for mitochondrial-derived superoxide and hydroxyl radicals when used in low micromolar concentrations, but at higher concentrations, the probe leaks into the cytosolic space and reports on increases in ROS in both the mitochondria and the cytosol [110]. The increase in NQO2-mediated ROS measured with MitoSox may suggest that the ROS was of mitochondrial origin. Given the complexities of cellular ROS production and signalling, additional research is required to determine whether NQO2 produces ROS directly or if NQO2 regulates cellular processes to shift metabolism towards oxidative phosphorylation, thereby increasing ROS indirectly.

**Table 2:** NQO2-directed production of reactive oxygen species (ROS) from the literature.

Study description	Cell system	Co-substrate added?	Method for specifying if ROS is NQO2-specific	Additional treatment?	ROS detection method	Ref
Showed that there was NQO2-assoiciated ROS in injured rat carotid artery	Injured rat carotid artery	No	NQO2 genetic knockdown	N/A	H_2_DCFDA^1^	[38]
Treatment with the listed oxidative insults caused NQO2-associated ROS as NQO2 inhibition by S29434 decreased ROS	U373 cells, human glioblastoma astrocytoma	No	S29434^2^	Paraquat, rotenone, hydrogen peroxide	MitoSox^2^, Dihydroethidium^3^, Aminophenyl fluorescein (APF)^4^, Malondialdehyde detection assay (MDA)^5^	[21]
Acetaminophen treatment increased ROS, but NQO2 inhibition with the listed inhibitors and NQO2 genetic knockdown decreased ROS.	HeLa cells, human cervical cancer	No	Quercetin, resveratrol and imatinib. NQO2 genetic knockdown	Acetaminophen	MitoSox^2^	[109]
Menadione treatment increased NQO2-associated ROS as NQO2 inhibition with S29434 decreased ROS	Mouse oocytes	No	S29434^2^	Menadione	H_2_DCFDA^1^	[106]
Basal levels of ROS were NQO2-dependent as NQO2 inhibition decreased ROS. Although cells were treated with BNAH it did not seem to stimulate NQO2-associated ROS.	HEK 293 T cells, human embryonic kidney	BNAH at 100 μM	S29434^2^	N/A	H_2_DCFDA^1^	[15]
Treatment with the listed reagents led to NQO2-associated ROS as NQO2 genetic knockdown decreased ROS.	A549 and H1299 cells, lung cancer cell lines	No	NQO2 genetic knockdown	Curcumol, TRAIL	Dihydroethidium^3^	[42]
Treatment of the cells with BNAH and adrenochrome increased ROS signal in both the assays in an adrenochrome-concentration dependent manner. NQO2 inhibition decreased ROS signal. DNA comet tail assay showed that there was DNA damage upon adrenochrome treatment.	HT-22 cells, mouse hippocampal cells	BNAH at 100 μM	S29434^2^	Adrenochrome	CellROX Green^6^, H_2_DCFDA^1^,	[107]
Treatment with 6-OH-DA increased ROS signal compared with basal levels. Treatment with NQO2 inhibitor, S29343 decreased ROS signal.	Astrocyte cells	No	S29434^2^	6-OHDA, 6-hydroxydopamine	H_2_DCFDA^1^, MitoSox^2^	[23]

1 Dichlorodihydrofluorescein is sensitive to a wide variety of ROS molecules.

2 MitoSox measures mitochondrial hydroxyl and superoxide molecule.

3 Dihydroethidium measures hydroxyl and superoxide molecule.

4 Aminophenyl fluorescein (APF) reacts with a wide variety of ROS molecules.

5 MDA assay measures lipid peroxidation.

6 CellRox dye detects a wide variety of ROS molecules in live cells.

Enzymatic production of ROS (hydrogen peroxide and hydroxyl radicals) has been detected by EPR spectroscopy when menadione is reduced by purified NQO2 with NRH or 1-benzyl-1,4-dihydronicotinamide (BNAH) as reducing co-substrates [104]. In the same study, reduction in menadione by purified NQO1 (with NADPH as reducing co-substrate) also produced hydrogen peroxide and hydroxyl radicals. Previous research had shown by EPR that the two-electron-reduced quinol of menadione was unstable in solution and generated hydroxyl radicals by futile redox cycling, indicating a potential source for the ROS produced by enzymatic quinone reduction [111]. ROS production arising from menadione reduction was also demonstrated in cells using EPR spectroscopy. In this case, hydrogen peroxide formed as a result of NQO1 or NOQ2 catalytic reduction in menadione in CHO cells; however, in the case of NQO2, the synthetic reducing co-substrate BNAH had to be added exogenously [105]. Again, similar measurements were made with purified enzymes where ROS production by NQO1 and NQO2 was quantified after the addition of BNAH, NADH, and NADPH. For NQO2, only BNAH increased ROS formation, and the treatment of the NQO2-specific inhibitor, S29434 decreased ROS [12]. Similarly, it was also shown by EPR measurements that after treatment with menadione and BNAH, NQO2 produced large quantities of ROS, which could be inhibited by adding NQO2-specific inhibitors. The primary implication of the results of these experiments was that ROS may be generated because of NQO2 enzymatic action leading to non-enzymatic processes related to the chemistry of the quinol product. Another potential mechanism for direct ROS production by NQO2 could be redox cycling of the highly conserved copper metal cofactor of NQO2 [112–114] from Cu^+^ to Cu^2+^ by donating one electron to molecular oxygen and generating superoxide in the process.

## Conclusion

For all the literature on NQO2, its role in the cell remains opaque. NQO2 interacts with kinase inhibitors, antimalarial drugs, a variety of bioactive compounds and has been linked to learning and memory, changes in cellular metabolism, and cell viability. However, when NQO2 unexpectedly appears in a drug screen, proteomic or transcriptional analysis, it is often overlooked precisely because its potential cellular roles remain poorly defined. Our goal with this review was to provide a ‘birds eye view’ from a variety of research approaches that may provide clues as to the cellular function of NQO2 and opportunities for further investigation.

NQO2 evolved to be a very poor enzyme with the cellular redox couples NAD(P)H and this property is conserved across the amniotes, consistent with a selective pressure for this characteristic. On the other hand, NQO2 has retained a functional FAD cofactor and the ability to exist in an oxidized or reduced state. These two observations suggest a cellular role for NQO2 in redox sensing and regulation rather than catalytic reduction in electrophiles. Additional research is required to uncover direct links between NQO2 and specific signalling or metabolic pathways, and to further define molecular mechanisms underlying NQO2 function.

## Abbreviations

DMAT: 2-dimethylamino-4,5,6,7-tetrabromo-1H-benzimidazole
EIF: eukaryotic initiation factor
ER: endoplasmic reticulum
FAD: flavin adenine dinucleotide
NQO1: human quinone reductase 1
NQO2: human quinone reductase 2
NSCLC: non-small cell lung cancer
PDGFR: platelet-derived growth factor receptor
QR: quinone reductase
ROS: reactive oxygen species
TBBZ: 4,5,6,7-1H-tetrabromobenzimidazole
TNF: tumour necrosis factor
TRAIL: TNF-related apoptosis-inducing ligand
VSMCs: vascular smooth muscle cells
